# Climate Change, Tropospheric Ozone and Particulate Matter, and Health Impacts

**DOI:** 10.1289/ehp.11463

**Published:** 2008-07-10

**Authors:** Kristie L. Ebi, Glenn McGregor

**Affiliations:** 1 ESS, LLC, Alexandria, Virginia, USA; 2 The University of Auckland, Auckland, New Zealand

**Keywords:** air pollution, climate change, health impacts, ozone, particulate matter

## Abstract

**Objective:**

Because the state of the atmosphere determines the development, transport, dispersion, and deposition of air pollutants, there is concern that climate change could affect morbidity and mortality associated with elevated concentrations of these gases and fine particles. We review how climate change could affect future concentrations of tropospheric ozone and particulate matter (PM), and what changing concentrations could mean for population health.

**Data sources:**

We review studies projecting the impacts of climate change on air quality and studies projecting the impacts of these changes on morbidity and mortality.

**Data synthesis:**

Climate change could affect local to regional air quality through changes in chemical reaction rates, boundary layer heights that affect vertical mixing of pollutants, and changes in synoptic airflow patterns that govern pollutant transport. Sources of uncertainty include the degree of future climate change, future emissions of air pollutants and their precursors, and how population vulnerability may change in the future. Given these uncertainties, projections suggest that climate change will increase concentrations of tropospheric ozone, at least in high-income countries when precursor emissions are held constant, which would increase morbidity and mortality. Few projections are available for low- and middle-income countries. The evidence is less robust for PM, primarily because few studies have been conducted.

**Conclusions:**

Additional research is needed to better understand the possible impacts of climate change on air pollution–related health impacts. If improved models continue to project higher ozone concentrations with climate change, then reducing greenhouse gas emissions would enhance the health of current and future generations.

Extensive literature documents the adverse health impacts of exposure to elevated concentrations of air pollutants, particularly ozone, particulate matter with aerodynamic diameters <10 (PM_10_) and < 2.5 μm (PM_2.5_), sulfur dioxide, nitrogen dioxide, carbon monoxide, and lead. Worldwide in the year 2000, 0.8 million deaths and 7.9 million disability-adjusted life-years lost from respiratory problems, lung disease, and cancer were attributed to urban air pollution (World Health Organization 2002).

Because the state of the atmosphere at various scales determines the development, transport, dispersion, and deposition of air pollutants, there is concern that climate change could affect the burden of illness and mortality associated with these gases and fine particles. Therefore, we review studies projecting the impacts of climate change on air quality and studies projecting the impacts of these changes on morbidity and mortality, with a focus on studies published since 2000. We limited our review to the past several years because of significant advances in climate modeling (Solomon et al. 2007).

## Meteorology and Air Pollution

Air pollution concentrations are the result of interactions among local weather patterns, atmospheric circulation features, wind, topography, human activities (i.e., transport and coal-fired electricity generation), human responses to weather changes (i.e., the onset of cold or warm spells may increase heating and cooling needs and therefore energy needs), and other factors. Some locations, because of their general climate and topographic setting, are predisposed to poor air quality because the climate is conducive to chemical reactions leading to the transformation of emissions, and the topography restricts the dispersion of pollutants (Kossmann and Sturman 2004; Rappengluck et al. 1999).

Some air pollutants demonstrate clear seasonal cycles (Alvarez et al. 2000; Eiguren-Fernandez et al. 2004; Hazenkamp-Von Arx 2004; Kassomenos et al. 2003; Nagendra and Khare 2003). Certain weather situations provide the requisite meteorologic conditions for pollution episodes. Air pollution episodes are often associated with stationary or slowly migrating anticyclonic or high-pressure systems that reduce pollution dispersion, diffusion, and deposition (Rao et al. 2003; Schichtel and Husar 2001). The three-dimensional wind field, its related turbulence, and vertical temperature are important (McGregor 1999; Pal Ayra 2000). Meteorologic conditions also influence the chemical and physical processes involved in the formation of secondary pollutants such as ozone (Nilsson et al. 2001a, 2001b). Airflow along the flanks of anticyclonic systems can transport ozone precursors, creating the conditions for an ozone event (Lennartson and Schwartz 1999; Scott and Diab 2000; Tanner and Law 2002; Yarnal et al. 2001). Large-scale airflows not necessarily related to anticyclonic systems can interact with local topography, sea/lake and land breezes, or mountain and valley winds to increase pollutant concentrations (Cheng et al. 2001; Dayan and Levy 2002; Grossi et al. 1999; Hess et al. 2003; Kitada and Regmi 2003; Lennarston and Schwartz 2002; Liu and Chan 2002; Ma and Lyons 2003; Pillai and Moorthy 2001; Triantafyllou 2001). Distant weather systems such as tropical cyclones and low-pressure systems lying over coastal regions can lead to high pollution levels (Gallardo et al. 2002; Tanner and Law 2002; Wang and Kwok 2002).

Climate change could affect local to regional air quality directly through changes in chemical reaction rates, boundary layer heights (i.e., the layer of air near the ground that is affected by diurnal heat, moisture, and momentum transfer to/from the surface) that affect vertical mixing of pollutants, and changes in synoptic airflow patterns that govern pollutant transport. The synoptic scale corresponds to the typical size of mid-latitude high and low pressure systems (approximately a horizontal length of 1,000 km or 620 miles). Indirect effects could result from increasing or decreasing anthropogenic emissions via changes in human behavior or from altering the levels of biogenic emissions because of higher temperatures and land cover change. Higher temperatures can increase emissions of isoprene, a volatile hydrocarbon and ozone-precursor emitted by many woody plant species. However, establishing the scale (local, regional, global) and direction of change (improvement or deterioration) of air quality is challenging (Bernard et al. 2001; Rainham et al. 2001; Semazzi 2003; Swart et al. 2004). More is known about the potential impacts of climate change on ground-level ozone than on other air pollutants.

## Ozone

Ground-level ozone is a known pulmonary irritant that affects the respiratory mucous membranes, other lung tissues, and respiratory function. Exposure to elevated concentrations of ozone is associated with increased hospital admissions for pneumonia, chronic obstructive pulmonary disease, asthma, allergic rhinitis, and other respiratory diseases, and with premature mortality (e.g., Bell et al. 2004, 2007; Gryparis et al. 2004; Ito et al. 2005; Mudway and Kelly 2000). Outdoor ozone concentrations and activity patterns are the primary determinants of ozone exposure (Suh et al. 2000). Although a considerable amount is known about the health effects of ozone in Europe and North America, few studies have been conducted in other regions.

Ground-level ozone is both naturally occurring and, as the primary constituent of urban smog, a secondary pollutant formed through photochemical reactions involving nitrogen oxides and volatile organic compounds in the presence of bright sunshine with high temperatures [U.S. Environmental Protection Agency (EPA) 2007]. Land use changes over the past century affect ozone concentrations by altering vegetation patterns affecting biogenic volatile organic compound emissions that influence ozone production (Solomon et al. 2007). In addition, urbanization leading to heat islands can influence the local production and dispersion of ozone. In urban areas, gasoline-burning engines are major sources of volatile organic compounds, and nitrogen oxides are produced whenever fossil fuels are burned (U.S. EPA 2007). Temperature, wind, solar radiation, atmospheric moisture, venting, and mixing affect both emissions of ozone precursors and production of ozone (Mott et al. 2005; Nilsson et al. 2001a, 2001b). Because ozone formation depends on sunlight, concentrations are typically highest during the summer months, although not all cities have shown seasonality in ozone concentrations (Bates 2005). Observations show that trends in tropospheric ozone in the past few decades vary in sign, with increased or decreased ozone, and in magnitude in many locations, with significant upward trends at low latitudes (Solomon et al. 2007).

### Projected changes in tropospheric concentrations of ozone associated with climate change

There are two major sources of uncertainty when assessing the health impacts of future changes in tropospheric ozone concentrations: the extent of future changes in emissions of ozone precursors, and the degree to which future weather conditions could increase ozone concentrations. Future emissions are, of course, uncertain and depend on assumptions of population growth, economic development, regulatory actions, and energy use (Syri et al. 2002; Webster et al. 2002). Increased regulation of anthropogenic emissions of volatile organic compounds and nitrogen oxides from gasoline-powered engines means that biomass burning, including fires, will likely increase in importance as sources of ozone precursors. Assuming no change in the emissions of ozone precursors, the extent to which climate change affects the frequency of future ozone episodes will depend on the occurrence of the required meteorologic conditions (Hogrefe et al. 2004a; Jones and Davies 2000; Liao et al. 2006; Mickley et al. 2004; Murazaki and Hess 2006; Racherla and Adams 2006; Sousounis et al. 2002). Where climate change is projected to result in an increased frequency of stable anticyclonic conditions with little boundary layer ventilation and associated high temperatures, cloud-free conditions, and large solar radiation inputs, exceedance of current air quality standards will likely occur (Hogrefe et al. 2004a; Mickley et al. 2004; Murazaki and Hess 2006; Sousounis et al. 2002).

Future air quality, especially at the local to regional level, will depend partially on concentrations of pollutants at the global scale. Concentrations of ozone have risen since preindustrial times because of increasing emissions of methane, carbon monoxide, and nitrogen oxides, and this trend is expected to continue over the next 50 years, based on projections of annual mean maximum concentrations (Prather et al. 2003). However, as many major cities propose to reduce vehicle-based emissions of pollutants, it is expected that urban concentrations of ozone will rise less rapidly or be reduced (Cifuentes et al. 2001; Metcalfe et al. 2002). For example, it has been estimated that for the United States a 50% reduction of methane emissions would nearly halve the incidence of high ozone events (Fiore et al. 2002). Decreases in stratospheric ozone may also result in greater increases in ground level ozone in polluted regions because of an increase in ultraviolet radiation reaching the ground; ultraviolet radiation is involved in the formation of ozone (Solomon et al. 2003).

Changes in concentrations of ground-level ozone driven by scenarios of future emissions and/or weather patterns have been projected worldwide, with most projections for Europe and North America (Anderson et al. 2001; Aw and Kleeman 2003; Bell et al. 2007; Derwent et al. 2001; Hogrefe et al. 2004b, 2004c, 2005a, 2005b, 2006; Johnson et al. 2001; Leung and Gustafson 2005; Liao et al. 2006; Murazaki and Hess 2006; Racherla and Adams 2006; Steiner et al. 2006; Stevenson et al. 2000; Taha 2001; West et al. 2007); these studies, which range from global to local levels, are summarized in subsequent paragraphs. Although these studies are inconsistent in approaches taken and factors considered, most project increased tropospheric ozone concentrations, with high variability across regions. The Intergovernmental Panel on Climate Change concluded that climate change would modify a variety of chemicals and processes that control air quality, and the net effects are likely to vary from one region to another (Solomon et al. 2007).

On a global scale, if 1990 is treated as the reference period, then moderately high annual mean maximum ozone concentrations of 60 parts per billion (ppb) were projected for central Europe, China, Brazil, South Africa, and eastern North America during summertime (Anderson et al. 2001). By 2030, under a high emission scenario [Standardized Reference Emission Scenarios (SRES); see Appendix 1, A2 scenario], the area experiencing a background of 60 ppb was projected to expand significantly, especially in Europe and North America. By 2060, most of the populated continental areas would experience ozone concentrations of at least 60 ppb. By 2100, much of the Northern Hemisphere was projected to have annual mean maximum ozone levels of 60 ppb, as were most of the populated areas of the Southern Hemisphere (Anderson et al. 2001). However, Liao et al. (2006) and Racherla and Adams (2006), also using the SRES A2 scenario, projected that anthropogenic climate change could reduce the global ozone burden due to changes in atmospheric chemistry. Liao et al. (2006) also projected that surface ozone concentrations over or near populated and biomass-burning areas would increase.

Ozone concentrations were projected for 10 world regions in 2030, using a coupled general circulation model with interactive chemistry (LMDz-INCA; http://aoc.amma-international.org/researchProduct/aerosol-chemistry/lmdz/index.en.php?current=20060930) driven by the SRES A2 scenario (West et al. 2007). Modeled ozone concentrations for present conditions have been shown to reasonably agree with surface ozone measurements. The global average population-weighted 8-hr maximum ozone concentration was projected to increase by 9.4 parts per billion per volume (ppbv) compared with a simulation of the concentration in 2000, with the largest increases over South Asia (nearly 15 ppbv) and with large increases in the Middle East, Southeast Asia, Latin America, and East Asia.

Forkel and Knoche (2006) projected ozone concentrations in Germany under the IS92a “business-as-usual” scenario for the 2030s compared with the 1990s. Both biogenic volatile organic compound emissions and soil nitrous oxide emissions were projected to increase as temperatures rise. Projected daily maximum ozone concentrations increased by between 2 and 6 ppb (6–10%) across the study region; the number of days in the 2030s when daily maximum ozone exceeded 90 ppb increased nearly 4-fold, from 99 to 384.

Murazaki and Hess (2006), using the SRES A1 scenario and a global chemical transport model [MOZART-2; Model of OZone And Related chemical Tracers, version 2 (gctm.acd.ucar.edu/Mozart)], projected that by the end of the twenty-first century anthropogenic climate change alone would decrease background ozone concentrations over the United States, while ozone produced internally would increase. Over the western United States, the two forces approximately equaled each other. The authors projected that over the eastern United States, up to 12 additional days annually would exceed 80 ppbv.

Taha (2001) estimated increases in ozone concentrations by the end of the century in two large cities in California based on model results that linked output from two general circulation models to future emissions of nitrous oxides, volatile organic compounds, biogenic hydrocarbons, and sulfates, and air pollution models used to evaluate air quality compliance in these regions. Two frequently used attainment-demonstration modeling episodes were selected for the study, the 2010 projection of the 26–28 August “1987” episode for Los Angeles, and the 2005 projection of the 11–13 July “1990” episode for Sacramento Valley. Ozone concentrations on the last day of each episode were modeled. Under assumptions of future-year controlled emissions, the model suggested significant increases in ozone concentrations at the time of the base-case peak concentrations in the Los Angeles Basin (up to 26 ppb, an approximate 24% increase) and in the Sacramento Valley (up to 12 ppb, an approximate 10% increase). Aw and Kleeman (2003) simulated an episode of high air pollution in Southern California in 1996 with observed meteorology and then with higher temperatures. Ozone concentrations increased up to 16% with higher temperatures (+ 5°K); there was less consistency in PM_2.5_ response, depending on whether increased secondary particle formation or more evaporative losses from nitrate particles were more important. Steiner et al. (2006) reported variations across California in the sensitivity of ozone to changing temperatures, absolute humidity, biogenic volatile organic compound emissions, and pollution boundary conditions on a fine scale (4 km grid resolution).

In a coarse-scale analysis of pollution over the continental United States, Mickley et al. (2004) projected that, because of climate change alone (SRES A1b scenario), air pollution (as estimated by including combustible carbon monoxide and black carbon as tracers of anthropogenic pollution) could increase in the upper Midwest because of decreases between 2000 and 2052 in the frequency of Canadian frontal passages that clear away stagnating air pollution. Leung and Gustafson (2005) used regional climate simulations for temperature, solar radiation, precipitation, and stagnation/ventilation, and projected worse air quality in Texas and better air quality in the Midwest in 2045–2055 compared with 1995–2005. Bell et al. (2007) showed greater sensitivity of ozone concentrations in the Mid-Atlantic to changes in biogenic than to changes in anthropogenic emissions.

As part of the New York Climate and Health Project, Hogrefe and colleagues conducted local-scale analyses of air pollution impacts of future climate changes using integrated modeling (Hogrefe et al. 2004a, 2004b, 2004c; 2005a, 2005b) to examine the potential impacts of climate and land use changes on heat- and ozone-related health impacts in the New York City metropolitan area (Bell et al. 2007; Civerolo et al. 2007; Kinney et al. 2006; Knowlton et al. 2004). Hourly meteorologic data from the 1990s through the 2080s were simulated based on the SRES A2 and B2 scenarios. The global climate outputs were downscaled to a 36-km grid over the eastern United States using the MM5 (Penn State/National Center for Atmospheric Research Mesoscale Model 5; www.mmm.ucar.edu/mm5/) regional climate model. The MM5 results were then used as inputs to the CMAQ (Community Multiscale Air Quality) regional-scale air quality model. Five summers (June–August) in each of four decades (1990s, 2020s, 2050s, and 2080s) were simulated at the 36-km scale. Pollution precursor emissions over the eastern United States were based on U.S. EPA estimates at the county level for 1996. Compared with observations from ozone monitoring stations, initial projections were consistent with ozone spatial and temporal patterns over the eastern United States in the 1990s (Hogrefe et al. 2004a). Average daily maximum 8-hr concentrations were projected to increase by 2.7, 4.2, and 5.0 ppb in the 2020s, 2050s, and 2080s, respectively, because of climate change (Hogrefe et al. 2004b). The influence of climate on mean ozone values was similar in magnitude to the influence of rising global background by the 2050s, but climate had a larger impact on extreme values. When biogenic volatile organic emissions were allowed to increase in response to warming, an additional increase in ozone concentrations was projected that was similar in magnitude to that of climate alone (Hogrefe et al. 2004b). Climate change shifted the distribution of ozone concentrations toward higher values, with larger relative increases in future decades.

## Particulate Matter

PM is well known to affect morbidity and mortality (e.g., Dominici et al. 2006; Ibald-Mulli et al. 2002; Kappos et al. 2004; Pope et al. 2002), so increasing concentrations would have significant negative health impacts. Using a coupled climate–air pollution, three-dimensional model, Jacobson (2008) compared the health effects of preindustrial versus present-day atmospheric concentrations of carbon dioxide. The results suggest that increasing concentrations of CO_2_ increased tropospheric ozone and PM_2.5_, which increased mortality by about 1.1% per degree temperature increase over the baseline rate. Jacobson (2008) estimated that about 40% of the increase was attributable to ozone and the rest to PM. The estimated mortality increase was higher in locations with poorer air quality.

In comparison with ozone, assessments of the impact of climate change on other pollutants are few. These emphasize the role of local abatement strategies in determining the future concentrations of pollutants such as PM and sulfur dioxide and tend to project the probability of air quality standards being exceeded instead of absolute concentrations (Guttikunda et al. 2003; Hicks 2003; Jensen et al. 2001; Slanina and Zhang, 2004). The results vary by region. The severity and duration of summertime regional air pollution (combustion carbon monoxide and black carbon) episodes were projected to increase in the northeastern and midwestern United States by 2045–2052 because of climate change–induced decreases in the frequency of surface cyclones (Mickley et al. 2004). A U.K. study projected that climate change would result in a large decrease in days with high particulate concentrations due to changes in meteorologic conditions (Anderson et al. 2001). However, in the New York Climate and Health Project, PM_2.5_ concentrations were projected to increase with climate change, with the effects differing by component species, with sulfates and primary PM increasing markedly and with organic and nitrated components decreasing, mainly because of movement of these volatile species from the particulate to the gaseous phase (Hogrefe et al. 2005b, 2006).

Because transboundary transport of pollutants plays a significant role in determining local to regional air quality (Ansmann et al. 2003; Bergin et al. 2005; Buchanan et al. 2002; Chan et al. 2002; Claiborn et al. 2000; Martin et al. 2002; He et al. 2003; Helmis et al. 2003; Holloway et al. 2003; Jaffe et al. 2003, 2004; Kato et al. 2004; Kellogg and Griffin 2006; Liang et al. 2004; Moore et al. 2003; Ryall et al. 2002; Tu et al. 2004), changing patterns of atmospheric circulation at the hemispheric to global level are likely to be equally important as regional patterns for future local air quality (Langmann et al. 2003; Takemura et al. 2001).

## Potential Health Effects

Table 1 summarizes projections of morbidity and mortality based on current exposure–mortality relationships applied to projected ozone concentrations. An increase in ozone concentrations would affect the ability of regions to achieve air quality targets. There are few projections for cities in low- or middle-income countries, despite the heavier pollution burdens in these populations, presumably because of limited research funding.

The New York Climate and Health Project projected the potential health impacts of future ozone concentrations in the eastern United States (Bell et al. 2007; Knowlton et al. 2004). Knowlton and colleagues computed absolute and percentage increases in ozone-related daily summer-season deaths in the New York City metropolitan region in the 2050s compared with the 1990s (Kinney et al. 2006; Knowlton et al. 2004). The availability of county-scale ozone projections made it possible to compare impacts in the urban core with those in outlying areas. Increases in ozone-related mortality due to climate change ranged from 0.4 to 7.0% across 31 counties. Bell et al. (2007) expanded the analysis to 50 eastern cities and examined both mortality and hospital admissions. Average ozone concentrations were projected to increase by 4.4 ppb (7.4%) in the 2050s; the range was 0.8–13.7%. In addition, ozone red-alert days could increase by 68%. These changes were projected to result in an 0.11 to 0.27% increase in nonaccidental mortality and an average 0.31% increase in cardiovascular disease mortality.

Ozone concentrations for Los Angeles and San Diego in 2050 were projected under the SRES A2 emission scenario (Hwang et al. 2004). Using several estimates of the ozone exposure–response relationship, Hwang et al. projected that mortality and hospital admissions would together increase up to approximately 3.7%, with most of the projected increases < 1%, depending on the city and the health outcome.

As part of a U.K. assessment of the potential impacts of climate change, Anderson et al. (2001) used projected daily meteorologic parameters for each day through December 2099, driven by the Intergovernmental Panel on Climate Change IS92a (business as usual) scenario, for a single grid point representing the British Isles. A global three-dimensional chemistry model was used to calculate the influence of the projected increases in emissions of methane, carbon monoxide, and nitrogen oxides from human activities on the global distribution of ozone through the year 2100. The impact of climate change on increases in the frequency and severity of the meteorologic conditions that lead to summertime ozone episodes was projected to be reduced by changes in European emissions of ozone precursor species. When the authors assumed thresholds for the health effects of ozone, the increase in health effects due to ozone was relatively small. If no threshold was assumed, then ozone was projected to increase premature deaths by 10, 20, and 40% for the years 2020, 2050, and 2080, respectively.

Approximately 500,000 excess deaths were estimated for the year 2030 due to the impacts of changing ozone concentrations and population growth in 10 world regions under the SRES A2 scenario (West et al. 2007). The daily acute mortality coefficient per parts per billion per volume ozone was taken from a study of 95 cities in the United States (Bell et al. 2004). Assuming a low concentration threshold of 25 ppbv and taking into consideration recently enacted legislation to control zone precursors, an estimated 191,000 deaths would be avoided globally (0.2% of the projected total number of deaths in 2030). An estimated 458,000 deaths would be avoided (0.5% of the projected total number of deaths in 2030) if currently available emission control technologies were aggressively employed globally. Sensitivity analyses showed that the results were significantly affected by the threshold assumed and the daily acute ozone mortality coefficient used.

## Discussion

Poor air quality currently affects the health of millions of people. Climate change has the potential to increase harmful exposures to elevated concentrations of ozone and PM_2.5_ through changes in regional weather patterns. However, there is high uncertainty about future projections. Sources of uncertainty include not only future climate change but also future emissions of greenhouse gases, ozone precursors, and other pollutants, as well as how population vulnerability and activity patterns may differ in the future.

Because of the high uncertainty of the extent and effectiveness of future emissions reductions, most studies that projected the impacts of climate change on air quality focused on future climate change alone and held precursor emissions constant over future decades. Therefore, the focus was on examining the sensitivity of ozone concentrations to alternative future climates rather than on attempting to project actual future ozone concentrations. On the basis of a limited number of modeling studies, climate change is likely to increase ozone concentrations in high-income countries when precursor emissions are held constant, leading to increased morbidity and mortality. There is less certainty of the possible impact of climate change on fine particulate concentrations.

More stringent emissions controls for ozone, PM_2.5_, and other pollutants can be expected with the growing body of evidence of the adverse health impacts of these air pollutants. Therefore, the extent to which climate change affects air quality will depend partially on ongoing regulatory control of ozone and PM_2.5_. At the same time, population sensitivity may change because of medical advances and changes in risk factors.

The main public health responses to the projected health impacts of climate change are mitigation and adaptation. Adaptation is not an effective risk management strategy for poor air quality, because physiologic mechanisms to decrease susceptibility to ozone and other air pollutants are limited. Therefore, if improved model experiments continue to project higher ozone concentrations under a changing climate, rapid reductions of emissions from fossil-fuel burning are needed to protect the health of current and future generations. Evidence suggests that reducing current tropospheric ozone concentrations reduces morbidity and mortality, with significant savings in medical care costs (e.g. Ostro et al. 2006).

For relevant agencies and institutions to develop appropriate and timely responses, additional research is needed to reduce the uncertainties associated with projections of the health impacts of changing concentrations of ozone and PM due to climate change. Research is needed to better understand the impacts of future emissions pathways, climate change impacts on concentrations of fine particles and gases, how changing weather patterns could influence the frequency and severity of episodes of poor air quality, population sensitivity, and how these factors might interact. Increasing greenhouse gas emissions suggest that future air quality could decline without increased regulations and development and deployment of new technologies.

## Figures and Tables

**Table 1:** Projected impacts of climate change on ozone-related health effects.

Area	Health effect	Model	Climate scenario time slices	Temperature increase and baseline	Population projections and other assumptions	Main results	Reference
New York metropolitan region, United States	Ozone-related deaths by county	Concentration– response function from published epidemiologic literature. Gridded ozone concentrations from CMAQ.	GISS driven by SRES A2, downscaled using MM5. 2050s	1.6–3.2°C in 2050s compared with 1990s	Population and age structure held constant at year 2000. Assumes no change from U.S, EPA 1996 national emissions inventory and A2; consistent increases in NO_x_ and VOCs by 2050s.	A2 climate only: 4.5% increase in ozone- related deaths. Ozone elevated in all counties. A2 climate and precursors: 4.4% increase in ozone- related-deaths. (Ozone not elevated in all areas due to NO_x_ interactions.)	Knowlton et al. 2004
50 cities, eastern United States	Ozone-related hospitalizations and deaths	Concentration– response function from published epidemiologic literature. Gridded ozone concentrations from CMAQ.	GISS driven by SRES A2, downscaled using MM5. 2050s	1.6–3.2°C in 2050s compared with 1990s	Population and age structure held constant at year 2000. Assumes no change from U.S. EPA 1996 national emissions inventory and A2-consistent increases in NO_x_ and VOCs by 2050s.	Maximum ozone concentrations increase for all cities, with the largest increases in cities with currently higher concentrations; 68% increase in average number of days/summer exceeding the 8-hr regulatory standard, resulting in 0.11–0.27% increase in nonaccidental mortality and an average 0.31% increase in cardiovascular disease mortality.	Bell et al. 2007
Los Angeles and San Diego regions, California, United States	Ozone-related hospitalizations and deaths	Concentration– response function from published epidemiologic literature. Gridded ozone concentrations.	HadCM3 driven by SRES A2, downscaled using MM5, then a photochemical model (CAMx) in the 2050s and 2090s	2.1– 2.7°C in 2050s, and 4.6 to 5.5°C in 2090s	Population and age structure held constant. Assumes no change from U.S. EPA 1997 national emissions inventory and A2- consistent increases in NO_x_ and VOCs by 2050s and 2090s.	Average increase in ozone peaks of 2.0–3.2 ppb in the 2050s, and 3.1–4.8 ppb in the 2090s. Increases in maximum peak concentrations are 2- to 3-fold higher. Percent increase in daily mortality in the 2050s range from 0.08 to 0.46 depending on the exposure–response relationship. Increases in the 2090s are 0.12–0.69. Projected increases in hospital admissions are higher.	Hwang et al. 2004
England and Wales, United Kingdom	Exceedance days (ozone, particulates, NO_x_)	Statistical, based on meteorologic factors for high pollutant days (temperature, wind speed); projections of U.K. and northwest Europe urban traffic emissions of ozone precursors.	UKCIP scenarios 2020s, 2050s, 2080s.	0.57–1.38°C in 2020s; 0.89 –2.44°C in 2050s; 1.13–3.47°C in 2080s compared with 1961–1990 baseline	Population and age structure held constant.	Over all time periods, large decreases in days with high particulates and SO_2_; small decrease in other pollutants except ozone, which increases. If a threshold is assumed, then the increase in health effects due to ozone would be relatively small. If no threshold is assumed, then ozone is projected to increase premature deaths by 10, 20, and 40% for the years 2020, 2050, and 2080, respectively.	Anderson et al. 2001
10 world regions	Premature mortality from acute ozone exposure	Ozone–mortality coefficient from a study of 95 cities in the United States.	Coupled general circulation model with interactive chemistry (LMDz-INCA) driven by SRES A2 for 2030.	Baseline simulated for 2000	Population growth and emissions under SRES A2. One realization included recently enacted legislative to control ozone, and another assumed maximum feasible reduction of ozone precursors.	Large increase in ozone in 2030 under the A2 scenario; global population–weighted 8-hr ozone increased 9.4 ppbv. Along with population growth, this was associated with approximately 500,000 additional deaths. Using a threshold of 25 ppbv, 191,000 deaths worldwide could be avoided using currently enacted legislation, and 458,000 deaths could be avoided using maximum feasible reduction technologies.	West et al. 2007

Abbreviations: CMAQ, Community Multiscale Air Quality; GISS, Goddard Institute for Space Studies; HadCM3, one of the climate models from the Hadley Centre, United Kingdom; NO_x_, nitrogen oxides; UKCIP, United Kingdom Climate Impacts Programme; VOC, volatile organic compound. Modified from Confalonieri et al. (2007).

## Appendix 1: SRES

SRES were developed by the Intergovernmental Panel on Climate Change as alternative images of how the future might unfold (Nakicenovic et al. 2000). Four different narrative storylines were developed to describe the relationships between the driving forces of greenhouse gas emissions and their evolution. Probabilities or likelihood were not assigned to the individual scenarios. There is no single most likely, or best guess, scenario. None of the scenarios represents an estimate of a central tendency for all driving forces or emissions.

Each SRES storyline assumes a distinctly different direction for future development, such that the four storylines differ in increasingly irreversible ways. The storylines were created along two dimensions: global versus regional development patterns and whether economic or environmental concerns would be primary. It is important to note that the scenarios do not cover all possible future worlds. For example, there is no SRES world in which absolute incomes are constant or falling. The A2 and B2 storylines are frequently used in modeling health impacts.

The A2 storyline describes a heterogeneous world with an underlying theme of self-reliance and preservation of local identities. Fertility patterns across regions vary slowly, resulting in continuously increasing global population. Economic development is primarily region oriented, and per capita economic growth and technological change are fragmented and slower compared with the other scenarios.

The B2 storyline describes a world in which the emphasis is on local solutions to economic, social, and environmental sustainability. It is a world with continuously increasing global population (at a rate slower than that of A2), intermediate levels of economic development, and less rapid and more diverse technological change.

The total cumulative CO_2_ emissions are categorized as very high in the A2 scenario [>1,800 GtC (gigatons)] and medium-low in the B2 scenario (1,100–1,450 GtC) in 2100.
